# Graphene-based nanotechnology in the Internet of Things: a mini review

**DOI:** 10.1186/s11671-024-04054-0

**Published:** 2024-07-02

**Authors:** Sharmi Ganguly, Joydip Sengupta

**Affiliations:** 1 Department of Electronics & Communication Engineering, Meghnad Saha Institute of Technology, Kolkata, 700150 India; 2 Department of Electronic Science, Jogesh Chandra Chaudhuri College, Kolkata, 700033 India

**Keywords:** Graphene, Internet of Things, Sensors, Communication systems, Energy storage

## Abstract

Graphene, a 2D nanomaterial, has garnered significant attention in recent years due to its exceptional properties, offering immense potential for revolutionizing various technological applications. In the context of the Internet of Things (IoT), which demands seamless connectivity and efficient data processing, graphene's unique attributes have positioned it as a promising candidate to prevail over challenges and optimize IoT systems. This review paper aims to provide a brief sketch of the diverse applications of graphene in IoT, highlighting its contributions to sensors, communication systems, and energy storage devices. Additionally, it discusses potential challenges and prospects for the integration of graphene in the rapidly evolving IoT landscape.

## Introduction

The advent of graphene, a two-dimensional material with extraordinary properties, has catalyzed a transformative shift within the Internet of Things (IoT), offering promising solutions to challenges such as energy consumption, data processing, and device miniaturization [1]. Graphene’s unique composition-a single layer of carbon atoms arranged in a hexagonal lattice-endows it with unparalleled electrical, mechanical, and optical properties, rendering it an optimal choice for diverse IoT components [2]. Its remarkable electrical conductivity, transparency, and flexibility empower it with the ability to facilitate ultrasensitive chemisensing capabilities crucial for environmental monitoring, healthcare diagnostics, and security protocols. Additionally, graphene serves as a cornerstone for advancing neuromorphic computing systems [3, 4], owing to its ability to emulate the synaptic connections present in biological neural networks, thus paving the way for efficient and adaptable IoT data processing. Furthermore, straintronic graphene devices [6, 7] exhibit promising potential for hardware security applications [8], leveraging the unique electronic properties of graphene to enhance data encryption and device authentication within IoT ecosystems. As graphene-based nanotechnology permeates various facets of IoT systems, including sensing, energy storage, and communication, its superior performance attributes continue to drive innovation, offering enhanced sensitivity, energy efficiency, and security features that propel the seamless integration and advancement of IoT networks.

In addition to its transformative potential in conventional IoT applications, graphene also holds promise for addressing environmental sustainability challenges within IoT ecosystems [9]. Its exceptional properties enable the development of energy-efficient solutions that minimize the environmental footprint of IoT devices and networks. Graphene's ability to enhance energy storage efficiency and optimize power consumption contributes to the advancement of sustainable IoT technologies [10]. Moreover, its compatibility with renewable energy sources further reinforces its role in promoting eco-friendly IoT systems. By leveraging graphene-based innovations, such as efficient energy harvesting and storage, IoT networks can operate more sustainably, aligning with global efforts towards green technology adoption and environmental conservation.

The intersection of IoT and graphene technology is discussed in the review paper, with their combined potential for revolutionizing various industries highlighted. The facilitation of seamless connectivity among devices by IoT, enabling advanced data collection, sharing, and processing, is explored. Emphasis is placed on graphene's remarkable electrical conductivity and mechanical properties, positioning it as a pivotal material for next-generation electronics. A detailed scrutiny is conducted regarding graphene’s application in energy storage devices, communication systems, and sensor devices within the IoT framework. Furthermore, attention is drawn to the challenges associated with scaling up graphene production, underscoring the criticality of material quality for optimizing IoT device performance. Overall, the transformative capabilities of combining graphene with IoT technology are underscored by the review, while acknowledging the hurdles that must be overcome for widespread implementation.

## IoT and graphene

**Fig. 1:**
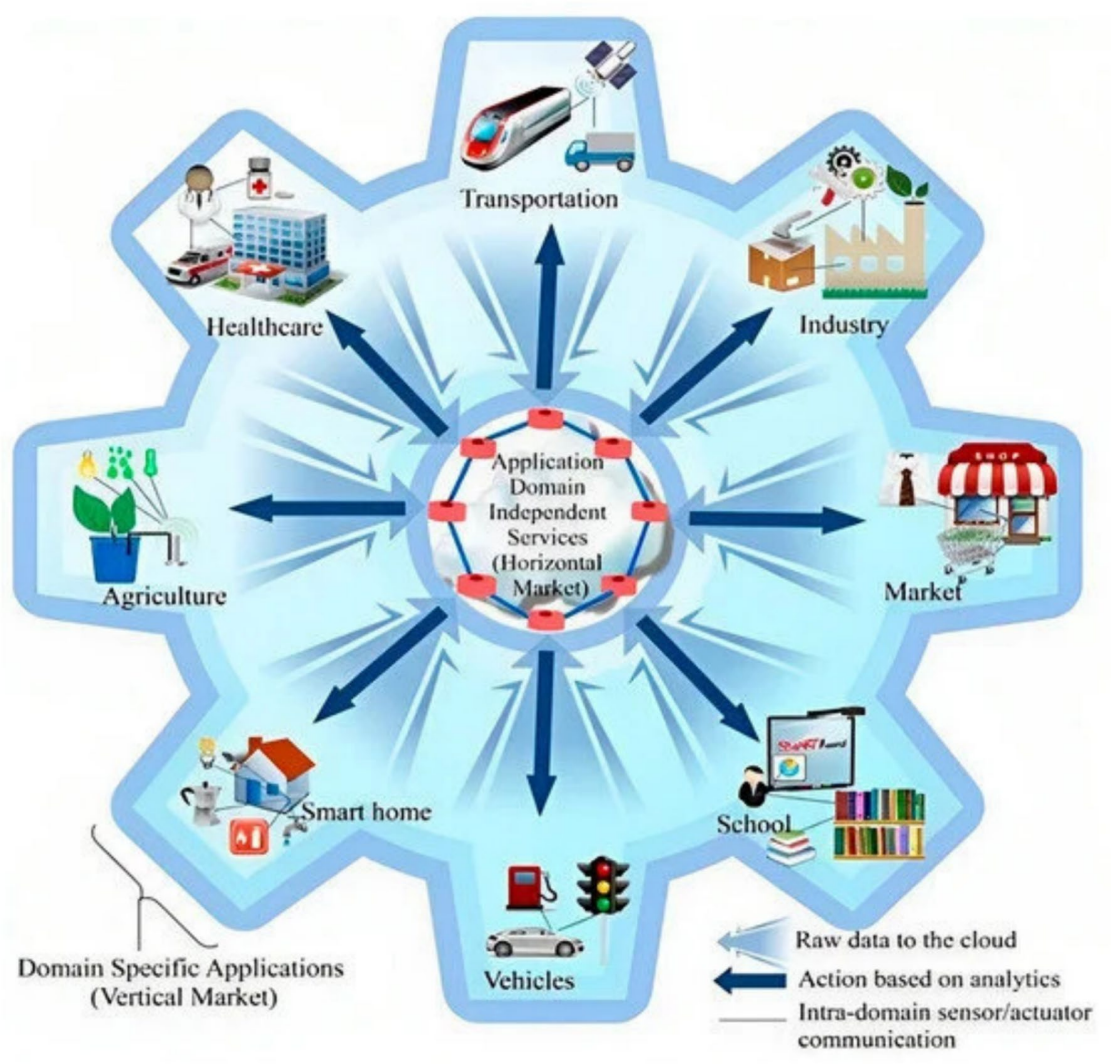
Schematic representation of IoT (Reproduced with permission from Ref. [11])

Through the effortless connection of gadgets and items equipped with a wide array of sensors and actuators, the IoT has brought about a significant shift in the environment of digital interfaces. This symphony opens up a world of unmatched opportunities and possibilities by enabling the self-organized collection, sharing, and advanced reasoning of information. The IoT has a pronounced effect on many industries, most notably from intelligent homes to large industrial complexes. IoT's cleverness in the context of smart homes is found in its ability to synchronize a variety of appliances, lighting systems, and security apparatuses harmoniously. This arrangement goes beyond simple utility since it gives these components the capacity to interact and respond in concert with the rhythm of human life. Meanwhile, in the industrial domain, IoT illuminates processes and mechanisms, endowing them with the cognitive ability to govern and optimize themselves. These self-adjusting modifications lead to increased production as well as higher efficiency than previously thought possible. Figure 1 shows the schematic representation of IoT in daily life schedules.

The initiation of cutting-edge sensor modules, emerging wireless networks, and contemporary cloud services enable the realization of IoT applications on a massive scale [12]. The data gathered by interacting with the environment through the sensing capabilities of IoT devices is transmitted over communication networks [13]. The number of sensing modules connected to the internet is growing at an accelerated rate; by 2030, it is anticipated to surpass 500 billion. The current sensor network technologies and standards are inadequate for applications involving large-scale interaction and sensing. It is difficult to construct intelligent IoT applications on a wide scale because of this [14]. Implementing energy-efficient functions in battery-operated IoT platforms can prolong network life without requiring device replacement or battery recharging [15]. IoT sensing applications create vast amounts of continuous data, and during energy-intensive operations and data transfer, restricted sensor modules may result in bottlenecks [16]. Examples of these applications include multimedia ones that make use of IoT and dense sensor networks. Wireless sensor networks that are connected to the internet through dense deployment of devices are used to send multimedia data, including photos, videos, and sounds, from the physical environment [17].

An exquisite performance of automation results from this symphonic arrangement of cognitive capabilities and connectivity inside the IoT paradigm. By increasing automation, machines and processes may operate more efficiently and without the need for human intervention. This in turn sparks a new era of data-driven decisions, meaning that an informed precision-driven environment is sustained. These disruptive dynamics are immediately apparent in a variety of fields, such as manufacturing, where predictive maintenance is becoming a reality and reducing downtime by extending the lifespan of machinery, or healthcare, where IoT-mediated devices transmit real-time patient metrics to clinicians, revolutionizing diagnostics and care.

**Fig. 2:**
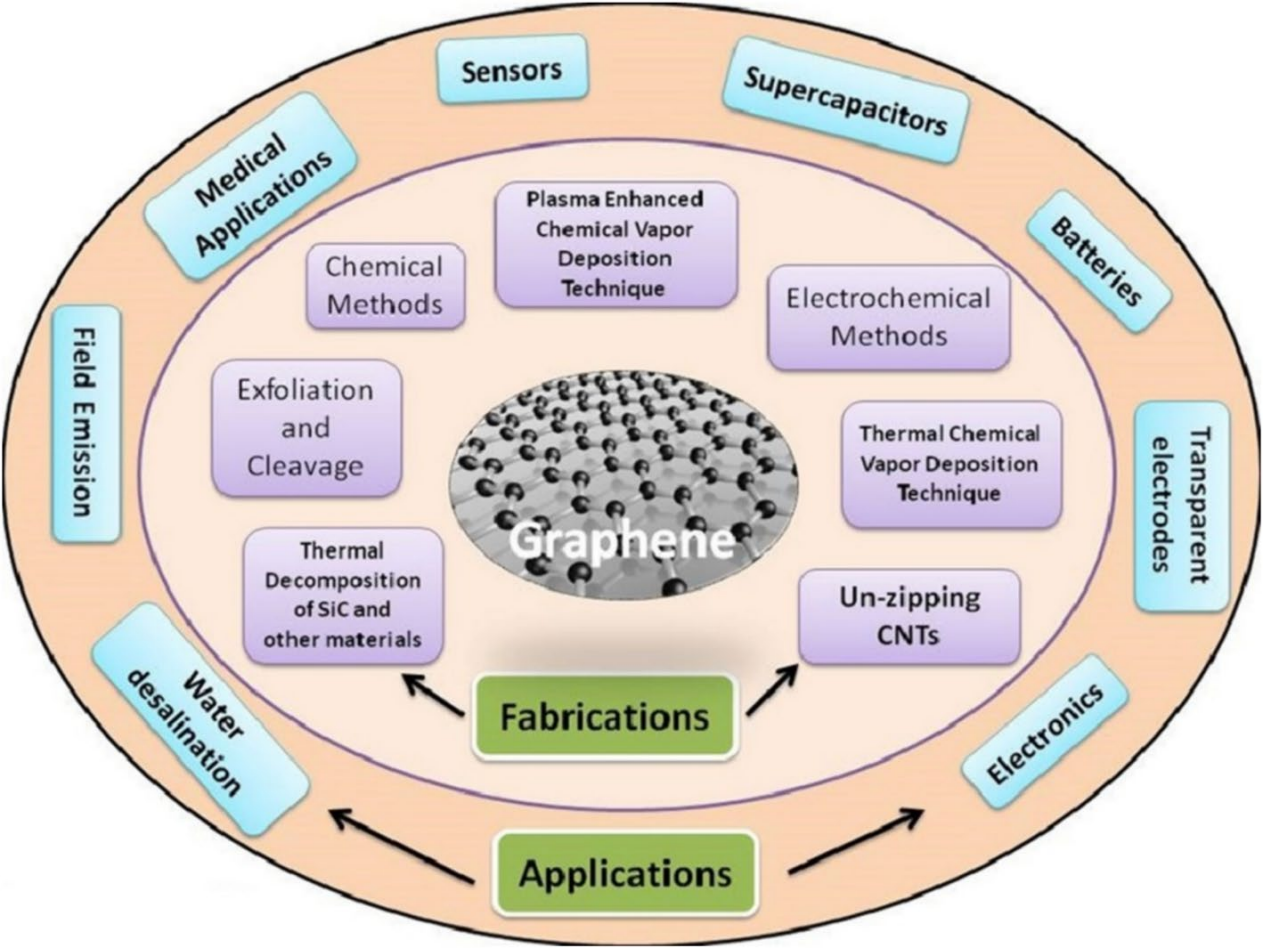
Synthesis and applications of Graphene (Reproduced with permission from Ref. [25])

Graphene, with its unmatched electrical conductivity, stands out as an unquestionable virtuoso in this complex embroidery. Due to its remarkable properties, graphene, a planar sheet of sp^2^-bonded carbon atoms densely packed in a honeycomb crystal lattice and only one atom thick, has garnered a lot of interest as a possible material for next-generation electronics [18, 19]. Graphite was originally converted to graphene by a process known as micro-mechanical cleavage [20]. This method made it simple to produce high-quality graphene crystallites and sparked a flurry of experiments [21]. With an astonishingly low absorption ratio of 2.3% of white light, intrinsic graphene is classified as a zero-gap semiconductor due to its special electrical properties, which also result in an opacity that is surprisingly high for an atomic monolayer [22]. At room temperature, electrical characterization has revealed extraordinarily high electron mobility, with experimentally reported values exceeding 15,000 cm^2^V^−1^ s^−1^ [23]. The graphene sheet's resistance would be 10^−6^ Ω-cm, which is less than silver, used as the substance with the lowest resistivity known to exist at ambient temperature [24]. The outstanding electrical properties of graphene have drawn interest in applications for circuit components, sensors, transistors, field emitters, and conducting electrodes for future electronics. Graphene can be used as the channel in a field effect transistor (FET) because of its high electron mobility and low Johnson noise. This unique property confirms that electric charge moves smoothly through the complex network of IoT devices, putting graphene at the forefront of charge transport technologies. Parallel to this, the complex hexagonal arrangement of carbon atoms in graphene ensures that data flows freely, protecting the vital component of digital communication from deterioration or loss. Different synthesis procedures and applications are shown in Fig. 2. Beyond its extraordinary electrical qualities, graphene’s mechanical robustness and flexibility create ground-breaking opportunities for IoT design.

The IoT’s structural underpinnings are robustly supported by this one substance, which guarantees the system's resilience against obstacles. Furthermore, the mechanical flexibility of graphene offers a way to design IoT structures that are flexible enough to evolve, meaning that robustness and flexibility go hand in hand. This versatility also reaches the wearables space, where graphene offers an exceptional blend of robustness and ergonomic comfort, sending these personal gadgets to the pinnacle of user-centred design. Graphene's transcendence also reaches the domain of interfaces for vision and other senses. Because of its intrinsic transparency, it reimagines traditional touch screens, making it difficult to determine between the digital and tactile worlds and giving consumers an increasingly immersive experience. Protecting public health and maintaining ecological balance is made possible by the material's large surface area and accuracy in gas sensing, which are applied in the field of environmental monitoring. Graphene's benefit to hybrid structures is a further aspect of its influence. By utilizing graphene's special properties, these cooperative combinations advance energy storage technologies and provide gadgets with longer life spans, higher densities, and increased efficiency. Furthermore, in the field of biomedical applications, the incorporation of graphene into hybrid systems unleashes new creative possibilities, driving breakthroughs in drug delivery systems, implantable devices, and diagnostic instruments. In conclusion, an unprecedented period of creativity and progress is ushered in by the combination of graphene's remarkable properties and the paradigm-shifting potential of IoT. This intersection challenges our preconceived notions about what is possible in terms of technology and extends an invitation for us to join it on a voyage of discovery. When these two realms come together, they write a story of exploration and change those changes in lives, experiences, and industries.

## Graphene IoT-based applications

### Graphene IoT in energy storage devices

Wearable technology and roll-up displays are two examples of the highly anticipated portable and bendable electronics of the recent past. Requiring compatible battery packs with high energy and power density is necessary to properly realize the functionality of these gadgets in a bendable shape. However, the dearth of trustworthy materials combining high mechanical flexibility, superior electrical conductivity, and good stability in electrochemical conditions makes the creation of bendable power sources extremely difficult. Pushparaj et al. [26] suggested that the fundamental components for creating different flexible, hybrid, dual-storage battery-in-supercapacitor devices are nanoporous cellulose paper embedded with aligned carbon nanotube electrodes and electrolytes. Lithium-ion (Li-ion) batteries have been manufactured in diverse ways to increase their flexibility. These methods include the use of active materials, and electrolytes, which are then combined to create flexible electronic devices that, when compared to conventional Li-ion batteries that are not flexible, have promising electrochemical properties. This research was conducted by Choi and his team [27].

Being chemically stable, carbon-based materials are frequently utilized in fuel cells, supercapacitors, and batteries over a broad range of electrochemical potentials. A novel class of two-dimensional carbon nanostructures called graphene nanosheets has garnered a lot of interest among the other carbon nanostructures. V Suryanarayanan [28] and his group describe a variety of carbon materials, ranging from amorphous to highly orientated graphitic materials, and the methods used to characterize the lithium insertion/de-insertion process. Their exceptional qualities such as their great mechanical strength, low weight, superior electronic transport abilities, and high surface area to volume ratio-are mostly to blame for this. According to Geim et al. [24] and co-workers, graphene-based micrometer-sized sensors can identify distinct events such as a gas molecule adhering to or separating from the material’s surface.

Recent research has demonstrated that graphene nanosheets may be readily produced in huge numbers by chemically converting affordable, commercial graphite. Graphene paper is a macroscopic, paper-like substance made of graphene nanosheets that can be produced affordably using this manufacturing technique. Graphene paper, in particular, performs better than many other materials that resemble paper, such as carbon nanotube paper, in terms of mechanical properties. By reducing a colloidal suspension Stankovich et al. [29] were able to get the sheets to aggregate and subsequently produce a high-surface-area carbon material made of thin graphene-based sheets of exfoliated graphene oxide sheets by dipping in water with hydrazine hydrate [30]. Colloidal suspensions of reduced graphene oxide sheets when produced as a paper material have strong electrical conductivity. Thus, it provides a method, as shown by S. Park et al., for producing a uniform aqueous suspension of chemically modified graphene [31].

Polymers are commonly utilized as a flexible substrate for flexible devices. However, polymers do not provide easy electrode production since they break down quickly at relatively low temperatures, and commercially available cathode materials are formed at high temperatures. More intricate procedures are needed for newly emerging technologies like the transfer printing of inorganic compounds onto polymers. Additionally, during long-term battery cycling, poor adhesion with oxide materials might be troublesome [29, 32–36].

To address these issues, we recommend a hybrid electrode based on graphene, which is a novel method that provides superior adhesion and compatibility with the cathode substance. n actuality, graphene paper turned out to be a crucial element that functioned as both a conducting agent and a current collector. Graphene paper is a highly dependable material that may be employed in a variety of flexible shapes due to its exceptional qualities. High electrochemical activity should also be seen in the battery's active components, the cathode and anode, in every desired potential range. Using the pulsed laser deposition (PLD) method, a flexible electrode based on graphene was deposited to the cathode and anode. PLD is a well-known thin-film fabrication process that produces high-quality oxide ceramics at comparatively quick deposition rates. Even though large-scale deposits are still challenging, many engineers are working to increase the scalability. Therefore, we anticipated that flexible batteries could benefit from the PLD approach.

**Fig. 3:**
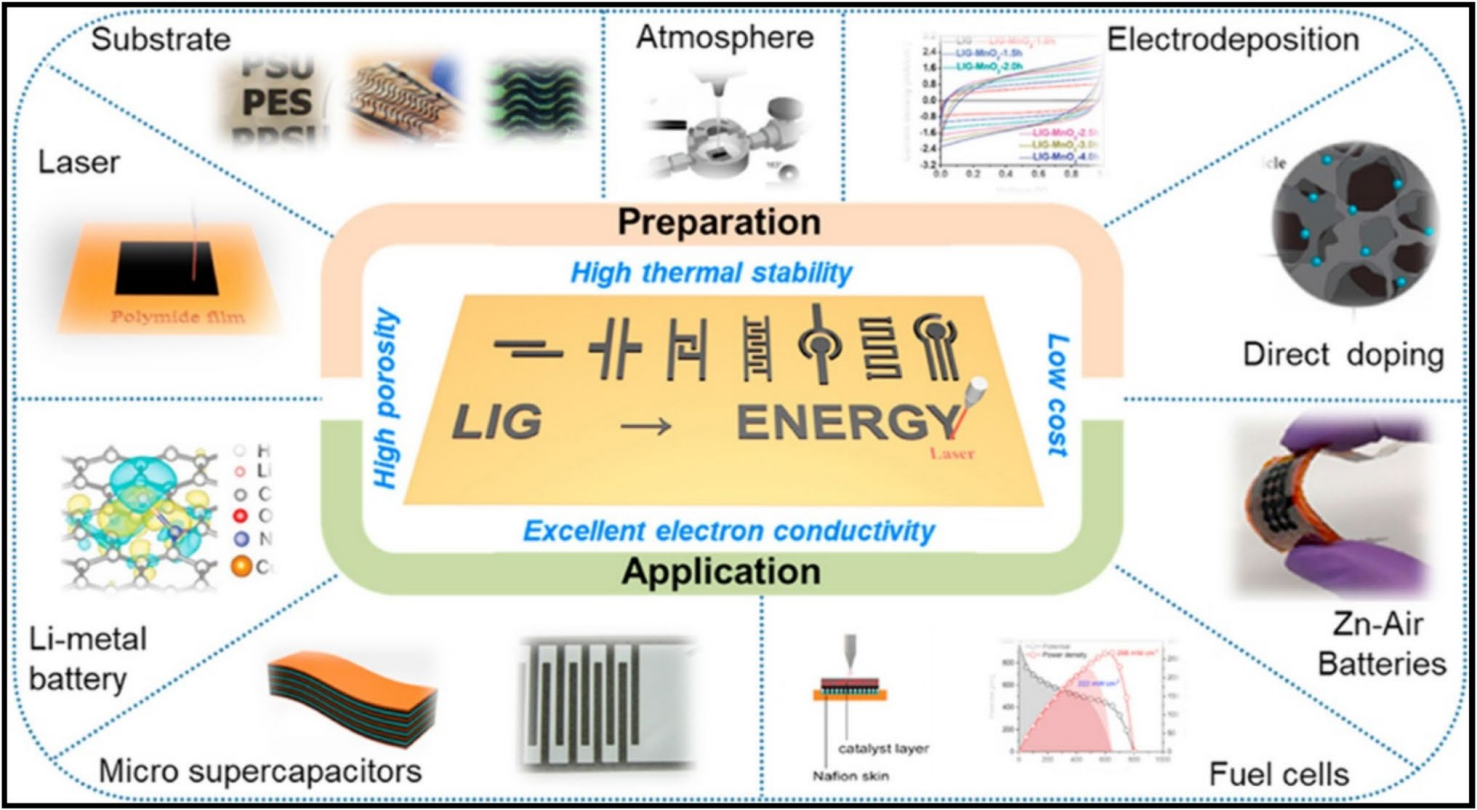
Laser-induced Graphene (LIG) preparation and applications (Reproduced with permission from Ref. [37])

**Fig. 4:**
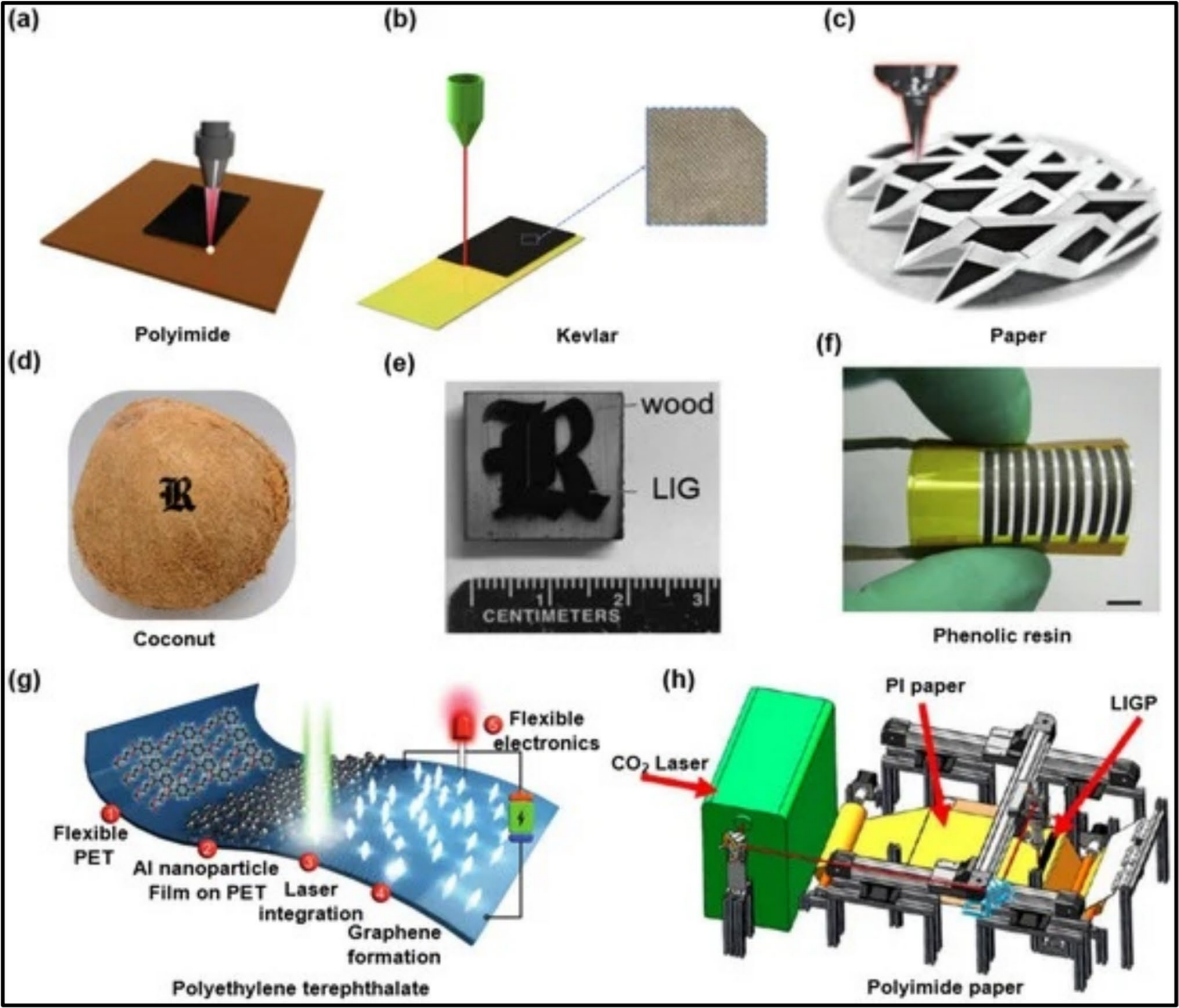
Fabrication of LIG on diverse substrates. **a** Polyimide film. Reproduced with permission from Ref. [38], **b** Aramid fibre fabric. Reproduced with permission from Ref. [39], **c** Paper soaked with the gelatin-mediated inks containing molybdenum ions. Reproduced with permission from Ref. [40], **d** Coconut shell. Reproduced with permission from Ref. [41], **e** Dry wood. Reproduced with permission from Ref. [41]. **f** Phenolic film. Reproduced with permission from Ref. [42]. **g** Polyethylene terephthalate/Al nanoparticle composite. Reproduced with permission from Ref. [43]. **h** Polyimide paper. Reproduced with permission from Ref. [44]

As seen in Fig. 3, laser-induced graphene (LIG) has garnered a lot of attention lately due to its numerous great features, including high porosity, high thermal stability, and superior electron conductivity. Figure 4 demonstrates the fabrication of LIG on different substrates.

### Graphene IoT in communication systems

The replacement of metals with lighter, less expensive, and energy-efficient materials is a major goal of modern technology. For structural applications in aviation and automotive, where the desire for increased efficiency and lower CO_2_ emissions is driving toward the use of carbon-based materials (such as carbon fibre composites and high-performance polymers), replacing up to 50% of the vehicle's metal parts, this “metal replacement” goal is already well-established. Because of the massive recent increase in electronic waste [45] and the need to lessen waste management issues related to heavy metals [46], which are not destroyed by incineration and will become air pollutants [47], metal replacement in "functional" applications, such as in cables or electronics, is equally important.

The inability to match the high conductivity of metals (> 10^7^ S/m) makes this task more difficult than structural applications. Though they have a high specific weight, high cost in thermal energy, and potential for oxidation and corrosion, copper, aluminium or silver offer good mechanical qualities and great electrical conductivity when formed into thin films. To produce the conducting circuits of the antenna, such metals are usually patterned and chemically etched during the fabrication of disposable antennas. The variety of substrates suitable for the fabrication of antennas is limited by the lengthy and demanding chemical etching process, which also involves a lot of polluting chemical agents and may damage the underlying substrate.

To replace conventional metal circuits with replaceable and ecologically friendly materials, new technological advancements would be needed. Using conductive ink solutions to print conductive patterns on rigorous or bendable surfaces is a viable method that can be scaled up for industrial use and has minimal manufacturing costs. This method makes it simple to achieve average conductivity, but it frequently calls for annealed printed inks at high temperatures to lower electrical resistivity.

**Fig. 5:**
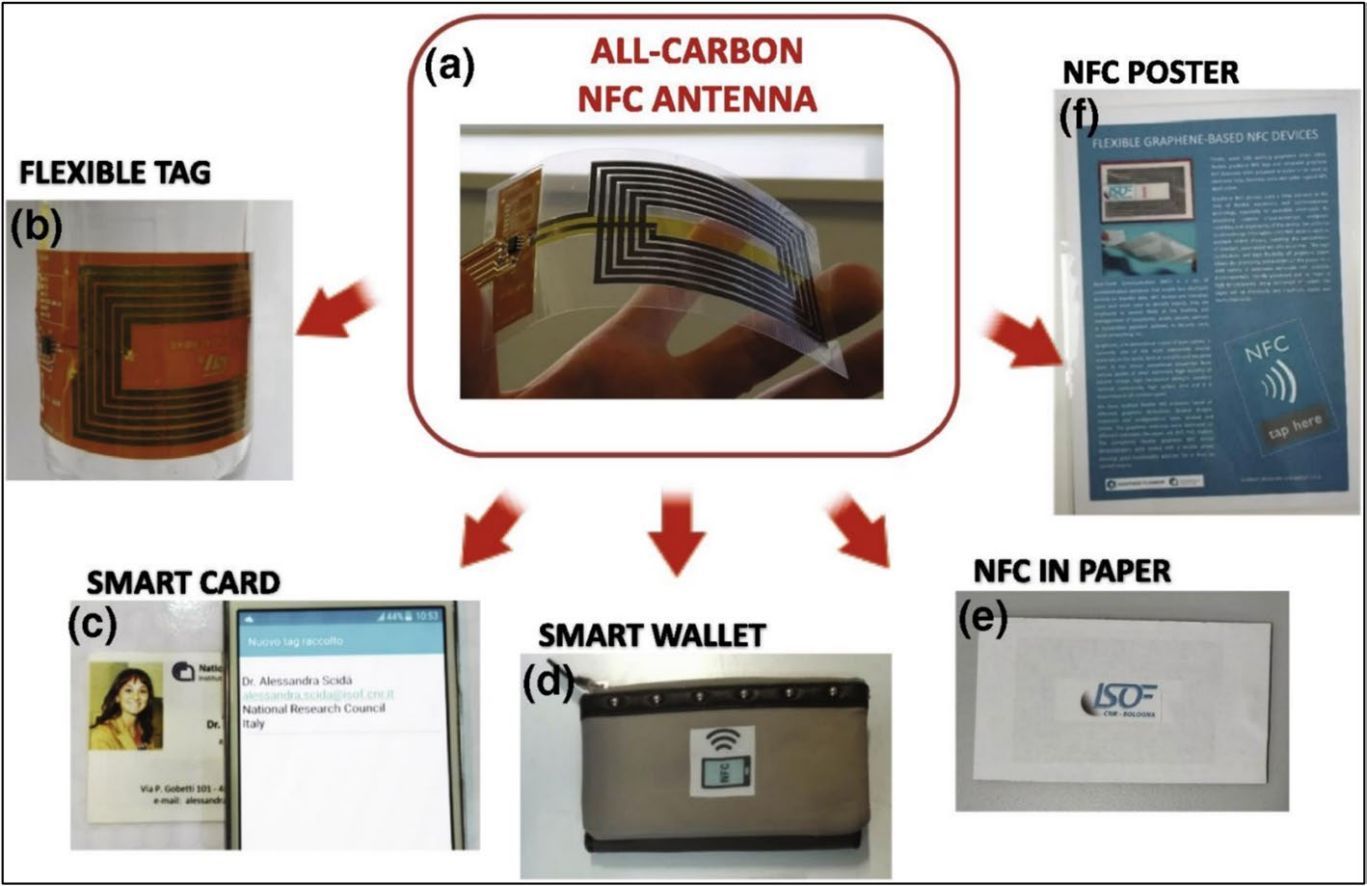
**a** Graphene-based flexible antenna and **b**–**e** Examples of different applications of the near-field applications (NFC) devices. (Reproduced with permission from ref. [48])

Annealing restricts applications on textiles and has the potential to deteriorate the fundamental polymeric substrate. Conducting inks used to manufacture antennas also commonly contain metallic nanoparticles. These particles can interact with oxygen or atmospheric water because of their large surface area, which accelerates the oxidation and degradation of the antenna compared to bulk metals. In innumerable applications, such as wearable electronics, antennas must be resistant to corrosion since they must endure hostile chemical environments. Graphene's high electrical conductivity enhances the performance of IoT communication systems, enabling faster and more reliable data transmission. A graphene-based nano-antenna is a tiny antenna structure made from graphene [48] which can operate across a wide range of frequencies, including terahertz [49, 50], due to graphene's tunable conductivity. At the nanoscale, their size facilitates precise signal control and manipulation of light and electromagnetic waves, while graphene's high electrical conductivity minimizes signal losses and maximizes efficient radiation, culminating in enhanced signal reception and transmission. Additionally, because graphene is compatible with CMOS technology [51], nano-antennas can be seamlessly integrated with current electrical components, opening the door to more compact and integrated Internet of Things communication systems, as shown in (Fig. 5).

The advancement of graphene exfoliation processes in recent years has made it possible to produce graphene-based products (graphene oxide, graphene solutions, etc.) at cheap cost and on a large scale. To create “graphene paper” (G-paper), which will have properties different from conventional, crystalline bulk graphite and have a high anisotropic 2-dimensional form and good processability, these graphene-based materials will be reassembled in thick, flexible layers [33].

Even though dipole antenna specifications are less stringent than those of typical mass-market consumer electronics products based on NFC protocols, researchers have already demonstrated that “graphene paper” may be used as an efficient conductor for antennas in their works [48, 52–55]. The carbon-based materials utilized in these most recent investigations have conductivities ranging from 102 S/m to 104 S/m, which is at least two orders of magnitude lower than the ideal graphite's 106 S/m [56]. The materials selected, the design of the device, and the production techniques allowed the antennas to completely comply with industry requirements while directly replacing metal-based antennas in radio-frequency identification (RFID) devices.

As previously mentioned, M. Kuscu and his colleagues [57] have shown that slow ligand-receptor binding processes, or DNA hybridization, on the receiver surface, produce a sizable amount of extra ISI. Consequently, once information molecules have been transferred and are attached to receptors for a longer period than a bit interval, the predicted received signal is deviated from, making it more challenging to detect succeeding bits. Dihydrolevoglucosenone (Cyrene), a non-toxic solvent, was employed by Kewen Pan et al. [58] to greatly accelerate and lower the cost of graphite's liquid phase exfoliation. The adjustable optical response of a recently suggested four-level ladder-type LQG subjected to two strong control fields and a weak probe field was reported by Azmat Iqbal Bashir [59]. For the safe exchange of quantum data through the Internet of Things quantum networking, they put out a quantum networking paradigm.

### Graphene IoT in sensor devices

An instrument called a "sensor" is used to measure a physical attribute and provide the results in an understandable format. More specifically, it's employed to detect any changes in the surroundings or an associated object. Sometimes, sensors are referred to as “transducers” based on the output signal. For sensors, the input and output signals are usually in the same formats; however, a transducer is a device that converts an output signal to an electrical signal. The major use of the Internet of Things is to connect these sensors and processors so they can communicate with one another intelligently. The IoT is made up of micro/nanosensors, actuators, and processors that are combined with tiny power supplies and wireless antennas. When an output signal is transformed into an electrical signal, it is referred to as a transducer. Another piece of equipment that regularly appears in the control system of the Internet of Things is called an actuator; its definition is sometimes mistaken for that of a sensor. Consequently, the Industrial Internet Consortium has included new definitions for “IoT Sensor” and “IoT Actuator” in its updated "dictionary" of technical words for the industrial Internet of Things. The energy forms employed or transduced during sensing constitute the basis for the technology in a sensor, and these energy forms are linked to certain observable qualities. Table 1 deals with the properties of graphene /graphene oxide-based sensors for gas sensing.

**Table 1:** Graphene/ graphene oxide sensors for different gas sensing properties

Sensor	Fabrication technique	Target gas	Concentration	Sensitivity	Gate voltage	Conclusion	Selectivity	Reference
Graphene channel Si substrate, back gate	Li_5_AlO_4_dielectric lithography free fabrication	NH_3_	1.5–3 ppm	20% at 3 ppm	− 2 to + 2 V	Low leakage and gate voltage, high breakdown voltage	–	[60]
Pd–Ag Graphene channel SiO_2_/Si substrate, back gate	Pd–Ag modified graphene Channel, MEMS platform	H_2_	50–50000 ppm	1.32% at 2000 ppm	0–4 V	Inbuilt micrometer, low power, poor sensitivity	–	[61]
TiO_2_-Graphene channel SiO_2_/Si substrate, back gate	TiO_2_ graphene hybrid channel	NH_3_	25 ppm	16.4% at 25 ppm	− 75 V to + 100 V	Excellent recovery, selectivity, and sensitivity substantial gate bias	Selectivity over acetone, ethanol, methanol	[62]
Graphene channel Si substrate, back gate	HfO_2_	NO_2_,	10 ppm	24% at CO_2_	− 5 V to + 5 V	Low gate voltage	–	[63]
Graphene channel SiO_2_/Si substrate, back gate	SiO_2_ treated with H_2_ annealing, Al_2_O_3_ coating, CF_4_RIE	O_2_	10–1000 ppm	25 V		Best sensitivity obtained for H_2_ annealing	–	[64]
Graphene channel SiO_2_/Si substrate, back gate	The ionic liquid top gate	CO_2_	0.4–16%	10% at 16% of CO_2_	− 0.8 V to + 0.8 V	Low gate voltage, Moderate Selectivity	Moderate Selectivity	[65]

#### Graphene-based piezo-electric and piezo-resistive sensors

**Fig. 6:**
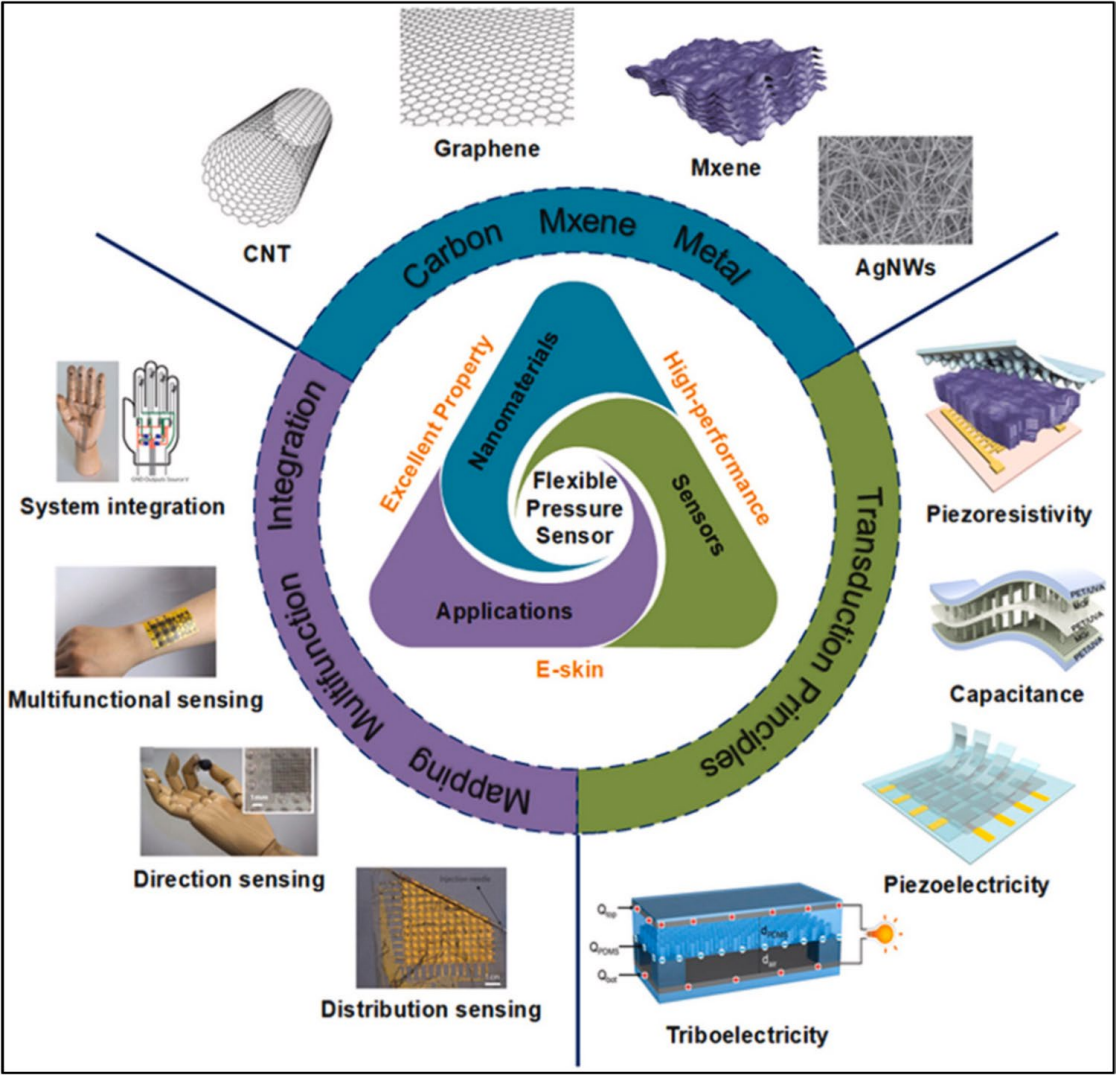
Flexible pressure sensor materials, the working principle and applications (Reproduced with permission from Ref. [71])

The piezoresistive effect is the change in resistivity that occurs when a material is subjected to a mechanical force. Conversely, a material produces a voltage when it is subjected to a mechanical force. Because the mechanical and electrical properties of a resistive network can be readily extracted by an analog-to-digital converter circuit, piezoresistive or piezoelectric sensors are simple to construct and read. Consequently, these sensors find widespread use in a wide range of applications, including acoustic transducers, high voltage generators, pressure sensors, and dynamically controlling material deformation through the application of an external electric force, among many others [66]. Figure 6 below illustrates the various materials utilized in flexible pressure sensors, as well as their functions and uses.

For sensing, actuating, and energy harvesting purposes, graphene has been utilized as a piezoelectric material in NEMS platforms. The centrosymmetric crystal structure of pristine graphene prevents it from exhibiting piezoelectricity in either the metallic/semi-metallic form or the semiconducting/dielectric form [67]. However, Wang et al. [68] attempted to produce a bi-axial strain—known as the band-piezoelectric effect—that results from a combination of electrostatic and piezoelectric actuation to create 2D piezoelectricity in a suspended few-layer graphene membrane. In a few-layer graphene on a Si_3_N_4_ substrate as well as a monolayer graphene membrane on a SiO_2_ grating substrate, Kholkin and coworkers [69, 70] reported strong piezoelectric effects. This is explained by the chemical interaction that results in polarization and the subsequent piezoelectric effect between the C-atoms of graphene and the O-atoms of the underlying SiO_2_ substrate as well as residual O-atoms in the amorphous Si_3_N_4_ layer.

Huang and coauthors showed that in graphene-based piezoelectric devices, opening up a band gap and changing the material's charge-transport properties—which may be essential for the material's use in future smart electronic devices—is not an effective way to control strain, in contrast to many CMOS devices, where strain can be easily controlled through device properties.

[72]. Graphene's high stiffness and low mass make it suitable for transducing motion in nano-electro-mechanical systems (NEMS) with a minimum detectable mass of 1.41 ± 0.02 zeptograms (10–21 g) at room temperature and a force resolution of 16.3 ± 0.8 aN Hz−1/2, as shown by Kumar and Bhaskaran [73]. This may result in the room-temperature benchtop single-molecule mass measurement of graphene [73].

Paper-based electronics have garnered a lot of attention lately because of their many advantages, including great flexibility, low cost, scalability, and ease of recycling or disposal. Consequently, graphene sensors based on paper are increasingly gaining popularity for a range of intelligent uses, such as environmentally friendly Internet of Things applications. To create piezoresistive sensors, several groups imprinted or directly coated media with graphene ink. These sensors have been successfully employed to gather a range of biophysical data, such as respiratory motion, voice recognition, and wrist pulse, for potential applications in motion detection and health monitoring because of their efficient performance in the low-pressure range within 20 kPa [74–78]. Graphene paper of superior quality has been produced by drop-casting a stable ink combination that was specially created. Through precise manipulation of the ethyl cellulose solvent blend's viscosity, the graphene/ethyl cellulose mixture may generate graphene paper covering on Kapton substrate at relatively large dimensions. The graphene study presented by Kamyar Karimi and colleagues [79] has the potential to be used in flexible electronics applications since the lowest resistivity and structure were obtained in a manner that was mostly independent of the mechanical loading range. Xinhui Wu and colleagues [80] achieved a flexible electrical and thermal conductor by functionalizing paper with conductive inks that were made by emulsifying graphene nanoparticles in isopropyl alcohol with carnauba wax. Ultimately, the compressed samples demonstrated a remarkable 200% increase in thermal diffusivity. The resultant flexible and multipurpose paper-based conductors may find use in thermally dissipative materials and electronics while also improving the environmental sustainability of the electronics industry. Karya gum is used as a bio-inspired exfoliation agent in Housseinou Ba's research group's environmentally benign and economical method of producing highly distributed few-layer graphene solution [81]. Table 2 contains the list of piezoelectric materials of different µm sizes and their different measurement quantities.

**Table 2:** Piezoelectric materials of different µm sizes and their different measurement quantities

Piezoelectric material (µm)	Substrate	Size (cm^2^)	Current (µA)	Voltage (V)	Power Density (mW/cm^3^)	Input frequency (Hz)	Reference
P(VDF-TrFE) 7 µm	PDMS	2 × 2	3.2	5.8 V	6.62	5–30	[82]
P(VDF-TrFE) 7 µm	CNT/PDMS	4 × 4	150 nA	1.4 V	0.018	NA	[70]
P(VDF-TrFE) 85 µm	MWCNT/PDMS	0.8 × 0.8	2.5	2.5 V	0.689	1–4	[83]
P(VDF-TrFE) 50 µm	ITO	1 × 1	2.6	4 V	2.08	1–100	[84]
P(VDF-TrFE) 30 µm	ITO and Gold Electrodes	1 × 1	1.88	4 V	2.5	1	[85]
PVDF 200 µm	Silicone	4.2 × 2	400nA	150 mV	3.57 × 10^−3^	1.4	[86]
PVDF 5 µm	PDMS	3 × 3	4nA	1.5 V	1.33 × 10^−3^	1–2.3	[87]

Experimental tests were conducted on a range of electrical applications involving the developed smart graphene paper, such as a lightweight conductive circuit that paves the way for the development of connected everyday objects and a flexible keyboard that can be directly connected to laptops. Again, wearable technology and Internet of Things applications are greatly impacted by the incorporation of sensors into clothing and fabrics. Rubber, nylon, wool, polyester, polyurethane, cotton, and other materials coated with graphene are utilized as temperature sensors, deformation sensors, antibacterial agents, pressure sensors, motion sensors and more [88–94]. The use of knitted flexible and wearable electronic platforms to incorporate smart devices in the Internet of Things technologies holds great potential.

#### Graphene-based chemical sensor

A chemical sensor is a device that selectively and reversibly reacts to a particular analyte by converting an input chemical quantity into an electrical signal that can be analysed. Both the sensing mechanism and the objects being sensed or detected can be used to categorize chemical sensors [95]. An active element absorbs and desorbs gas molecules or atoms, changing the electrical properties of the active element. This process is the basis of the gas-sensing mechanism. As a result, a key component of this sort of sensor device's great sensitivity is its high specific surface sites of the active elements [96]. Graphene's exceptional charge transport characteristics and extraordinarily large surface area make it an attractive choice for gas sensing applications. In terms of environmental safety, many hazardous gases are essential for sustainable applications in IoT networks of smart cities. There has been a lot of interest in gas sensing applications for study (both theoretical and experimental) due to graphene and its derivatives, as well as their nanocomposites and nanohybrids with metal nanoparticles, semiconducting metal oxides, inorganic materials, polymers, and doping [97, 98]. Another method for detecting the analytic gas in graphene-based gas sensors is the "optical" method, which measures and analyses the light (visible or infrared) absorption peak caused by different types of gas adsorption. While the majority of the gas sensors listed above are appropriate for monitoring the environment and other smart applications like the Internet of Things, they can also be intelligently employed for monitoring one's health. During natural disasters or unnatural calamities, the graphene-based gas sensor may find widespread and potentially life-saving applications in the tracking of trapped humans using their unique volatile chemical signature [99].

#### Graphene-based biosensors

In the healthcare sector, biosensing is another emerging chemical sensing technique [100], especially in Internet of Things applications. The integration of IoT into emergency healthcare services has led to a surge in the use of communicable biosensing technologies since it has made it possible to connect houses to smart hospitals via Electronic Health Records (EHR), which in turn allows for real-time symptom monitoring. There is a lot of interest in graphene-based biosensors because of their outstanding mechanical, optical, and electrical capabilities at room temperature, as well as their huge specific surface area that is both chemically and optically active. Because of these characteristics, graphene-based biosensors are perfect for biosensing applications that include the detection of a variety of biomolecules [101] and medications [102], including antigens, antibodies, and antibiotics. The biosensing mechanism is typically operated in two ways: electrically and optically. The most common technique for electrical biosensing of graphene is the electrochemical method. The aromatic domains and ionic groups on the surfaces of functionalized graphene interact with biomolecules through electrostatic interactions with positively charged molecules to produce detectable electrochemical signals [103]. Other electrochemical methods are the field-effect transistor (FET) based bio-detection method [104] and the impedimetric process, which use differences in electrical properties like conductivity and the Dirac point to derive the bio-recognition signal. The FET-based bio-detection method employs a suspended graphene layer between the drain and the source that is gated from either the front or the back. Various types of biosensors with their property details are listed in Table 3 below.

**Table 3:** Summary of biosensors

Type	Sensing mechanism	Measured property	Transducers used	References
Thermal detection	Bio-reaction results in the exothermic character	Heat of reaction or adsorption	Thermal	[105]
Resonant	Change of the viscosity and mass leads to the change of resonant frequency of the acoustic waves	Resonant Frequency	Mass sensitive	[106, 107]
ISFETs	The ionic analyte diffuses into the membrane hence changing the potential difference at the detecting interface	Surface potential	Ion selective membrane	[108–110]
Photometric	The change in refractive index of the solution leads to the change in the refractive angle of the incident light	Surface Plasmon resonance angle	Optical	[111, 112]
Electrochemical	Bio-reaction resulting in ions production or consumption will create transfer across the double layer of the transducer	Potentiometric, Amperometric, Impedimetric	Electrochemical	[113, 114]

Technological developments in graphene-based transistors have rendered them a promising option for post-silicon electronics [115]. There are many possible Internet of Things applications for graphene-based FET biosensors, thus it's vital to go over some specifics about them.

**Fig. 7:**
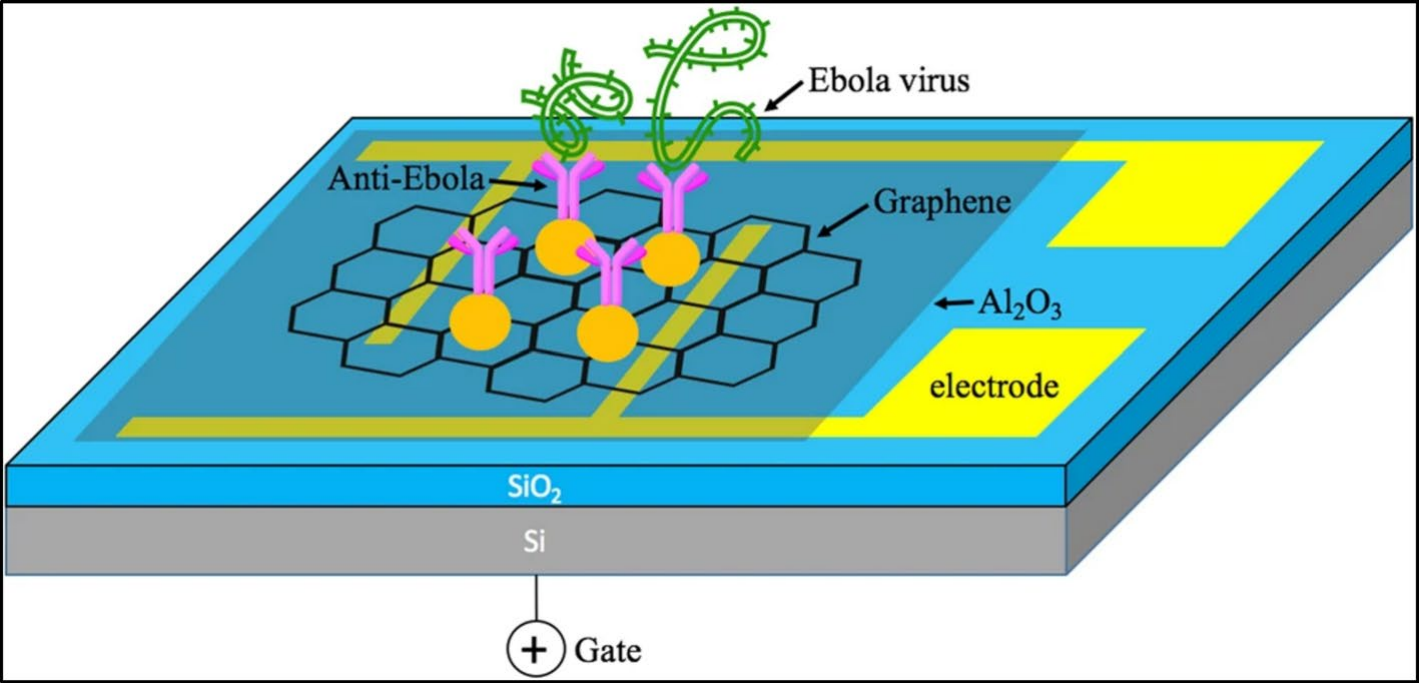
Field-Effect Transistor Biosensor for Rapid Detection of Ebola Antigen (Reproduced with permission from Ref. [118])

In a mono-to-few-layer graphene channel, an electrode is typically positioned on a dielectric and then suspended between the source and drain electrodes. The primary process driving fluorescence (FL)-based graphene biosensors is the FL quenching mechanism, which is exemplified by photo-induced charge transfer (PCT) between graphene (and its derivatives) and the target biomolecule [116] as well as fluorescence resonance energy transfer (FRET) from graphene derivatives to a quencher such as Au nanoparticles [117]. Using an immobilised Ebola antibody, Y. Chen and colleagues [118] designed and tested an rGO-based FET device to recognise the specific antigen displayed in Fig. 7. Gold nanoparticles (NPs) were electrosprayed onto the rGO sheet and subsequently chemically functionalized for antibody conjugation. An rGO sheet was positioned on the device to fill the space between the source and drain electrodes. Al_2_O_3_ was applied to the rGO sheet to produce surface passivation. Equipped with gold nanoparticles, Ebola antibodies functioned as sensing probes to identify Ebola antigens on the channel.

Recent reports have demonstrated the exceptional sensitivity and selectivity of graphene-based nanostructures as biosensors described in Table 4 below:

**Table 4:** Graphene-based Biosensor's current reports

Technique used	Receptor system	Detection limits	Target biomolecules	References
Graphene field effect transistor	Graphene/Tris–HCl	< 37.5 ng/L	Pb^2+^	[119]
Graphene field effect transistor	Graphene/anti-carcinoembryonic antigen	< 100 pg/mL	Carcinoembryonic antigen protein	[120]
Graphene field effect transistor	Graphene/DNA	163.7 ng/L	Pb^2+^	[121]
Graphene field effect transistor	Graphene	10 pM	DNA	[122]
Graphene oxide field effect transistor	Graphene oxide/pentacene	0.1 pM	Artificial DNA	[123]
Reduced graphene oxide field effect transistor	Urease/polyethylenimine/reduced graphene Oxide	1 μM	Urea	[124]
Reduced graphene oxide field effect transistor	Platinum nanoparticles	0.1 pM	Brain natriuretic peptide	[125]

## Challenges and future directions

The potential of graphene in the Internet of Things (IoT) is contingent upon achieving material homogeneity and quality. Its extraordinary features and distinct atomic arrangement, which provide IoT devices with revolutionary possibilities, are the very essence of its potential. But converting this promise into real benefits is fraught with difficulties stemming from the difficulties of manufacturing high-grade graphene at a scale commensurate with the vast requirements of Internet of Things applications. It is important to recognize the dynamic relationship that exists between material quality and device performance. Variations or irregularities in the graphene production process can have an impact on the whole range of IoT functionality, impairing device performance and dependability.

Present production methods, encompassing techniques such as chemical vapour deposition and exfoliation, stand as impressive feats of scientific ingenuity but face limitations in scalability and cost-effectiveness. This imperfection in large-scale graphene synthesis necessitates the quest for innovative methodologies that uphold not only the material’s innate properties but also the prerequisites of commercial viability. The successful integration of graphene into the IoT infrastructure necessitates novel pathways that address the gap between the promise of the material and the constraints of feasible manufacturing.

The integration process itself is a multifaceted conundrum, for graphene's intrinsic attributes, while extraordinary, are also inherently complex. Its two-dimensional nature, hexagonal lattice, and exceptional electrical conductivity bestow it with unique properties that, while transformative, pose challenges in harmonizing with established manufacturing processes and existing device architectures. Achieving seamless integration without perturbing the established frameworks, calls for a profound understanding of both the material's intricate characteristics and the complexities of IoT systems.

Yet, the challenges persist beyond the confines of manufacturing and integration. The environmental milieu wherein graphene-based IoT devices operate introduces its own set of challenges. Factors such as humidity, which may seem innocuous, have the potential to precipitate degradation in graphene-based devices over time. Addressing long-term stability becomes a paramount concern, necessitating innovative solutions that can uphold the performance of graphene-infused IoT devices under varying conditions. The absence of standardized fabrication methods and universally accepted metrics further exacerbates the situation. The pursuit of reliable and repeatable outcomes encounters stumbling blocks due to the dearth of established benchmarks and guidelines, fragmenting the progress in graphene-based IoT device development and rendering comparisons between studies a challenging endeavour. In the face of these formidable challenges, it is imperative to underscore the transformative potential that graphene holds for IoT. Its remarkable sensitivity positions it as a beacon of promise for advanced sensing applications within the IoT landscape. The development of graphene-based sensors, capable of detecting gases and biomolecules, presents the prospect of a paradigm shift in environmental monitoring and healthcare diagnostics. Its intrinsic electrical conductivity and substantial surface area render it an apt contender for energy harvesting and storage, and its high carrier mobility opens avenues for high-speed, energy-efficient wireless communication. Moreover, the realm of flexible and transparent electronics beckons with the potential to revolutionize wearables and displays. In the realm of healthcare, graphene's unique bio-interfacing capabilities could herald a new era in the monitoring and manipulation of biological processes, granting us unparalleled insights into the intricate tapestry of life. The journey towards realizing graphene's potential within IoT necessitates an exploration of hybrid materials that can augment its properties while addressing environmental impact concerns. While the path ahead may be fraught with challenges, the transformative potential of graphene-based nanotechnology within IoT is undeniable. It stands poised to reshape the landscape through advancements in sensing precision, energy solutions, communication efficacy, and healthcare innovations. As the scientific community forges ahead, it is the balance between aspiration and overcoming hurdles that will ultimately chart the trajectory of graphene's integration into the heart of IoT's technological evolution.

## Data Availability

Raw data is available upon request from the corresponding author.

## Code availability

Source code is available upon request from the corresponding author.
